# Genetic Analysis of Health-Related Secondary Metabolites in a *Brassica rapa* Recombinant Inbred Line Population

**DOI:** 10.3390/ijms140815561

**Published:** 2013-07-25

**Authors:** Hedayat Bagheri, Mohamed El-Soda, Hye Kyong Kim, Steffi Fritsche, Christian Jung, Mark G. M. Aarts

**Affiliations:** 1 Laboratory of Genetics, Wageningen University, Wageningen, 6700 AH, The Netherlands; E-Mails: mohamed.elsoda@wur.nl (M.E.-S.); mark.aarts@wur.nl (M.G.M.A.); 2 Department of Biotechnology, College of Agriculture, Bu-Ali Sina University, Hamedan 65174, Iran; 3 Faculty of Agriculture, Department of Genetics, Cairo University, Giza, Gamaa St. 12613, Egypt; 4 Natural Product Laboratory, Institute of Biology, Leiden University, Leiden, Sylviusweg 72, 2333BE, The Netherlands; E-Mail: h.k.kim@chem.leidenuniv.nl; 5 Plant Breeding Institute, Christian Albrechts University, Kiel, Olshausenstrasse 40, D-24098, Germany; E-Mails: steffi.fritsche@botany.ubc.ca (S.F.); c.jung@plantbreeding.uni-kiel.de (C.J.)

**Keywords:** *Brassica rapa*, secondary metabolites, nutritional value, Quantitative Trait Loci, tocopherol, glucosinolates

## Abstract

The genetic basis of the wide variation for nutritional traits in *Brassica rapa* is largely unknown. A new Recombinant Inbred Line (RIL) population was profiled using High Performance Liquid Chromatography (HPLC) and Nuclear Magnetic Resonance (NMR) analysis to detect quantitative trait loci (QTLs) controlling seed tocopherol and seedling metabolite concentrations. RIL population parent L58 had a higher level of glucosinolates and phenylpropanoids, whereas levels of sucrose, glucose and glutamate were higher in the other RIL population parent, R-o-18. QTL related to seed tocopherol (α-, β-, γ-, δ-, α-/γ- and total tocopherol) concentrations were detected on chromosomes A3, A6, A9 and A10, explaining 11%–35% of the respective variation. The locus on A3 co-locates with the *BrVTE1*gene, encoding tocopherol cyclase. NMR spectroscopy identified the presence of organic/amino acid, sugar/glucosinolate and aromatic compounds in seedlings. QTL positions were obtained for most of the identified compounds. Compared to previous studies, novel loci were found for glucosinolate concentrations. This work can be used to design markers for marker-assisted selection of nutritional compounds in *B. rapa*.

## 1. Introduction

*Brassica rapa* is a valuable source of health-promoting metabolites, like antioxidants, vitamins or glucosinolates. Plants, in general, produce an amazing diversity of low molecular mass natural compounds [1]. Over 100,000 metabolites have been detected [2], for which the structures of close to 50,000 have been elucidated [3]. Many of these compounds are part of secondary metabolic pathways, which are not directly involved in or essential for the central metabolic processes of the plant, but they play very important roles in ecological interactions, such as plant defense against pathogens and herbivores and the response to abiotic factors. This is in contrast to primary metabolites, such as carbohydrates, vitamins, amino and organic acids, which are found in all plants and are directly involved in normal growth, development and reproduction.

Plant secondary metabolites are of nutritional value for humans, due to their anticancer and health-promoting properties. Tocopherols, for instance, are essential nutrients that humans can only obtain via food. The α-, β-, γ- and δ-tocopherols produced by plants are jointly known as vitamin E. Seeds generally provide the bulk of tocopherols to the human diet [4,5]. They are lipid-soluble amphipathic molecules that can act as antioxidants [6–8]. Tocopherol content and composition can be determined accurately by High Performance Liquid Chromatography (HPLC) [9]. α-tocopherol is the most interesting component, as it is selectively taken up in the human liver, and its biological activity is 2–50 times higher than that of the others [4]. Generally, no β-tocopherol is found in *B. napus* and only very small amounts (<1%) of δ-tocopherol [10]. The average contributions of γ-tocopherol and α-tocopherol to total tocopherol in rape seed oil are 65% and 35%, respectively. The α- to γ-tocopherol concentration ratios in rapeseed vary from 0.32 to 1.40 mg kg^−1^, depending on growth condition and genotype [11]. Since γ-tocopherol has a ten-fold lower biological activity than α-tocopherol, increasing the α-tocopherol fraction is a potentially interesting breeding goal in order to improve the nutritional value of crop plants, like rapeseed [10] and *B. rapa*. Next to increasing α-tocopherol, to improve nutritional purposes, another interesting goal is to increase δ-and γ-tocopherol concentrations, to improve oil stability. γ-tocopherol is known to be the direct precursor of α-tocopherol [12]. The enzyme, γ-tocopherol methyltransferase (gTMT), catalyzes the conversion from δ- to β- or from γ- to α-tocopherol [13,14]. Some five to seven different quantitative trait loci (QTLs) with additive and/or epistatic effects have been mapped for γ-tocopherol and total tocopherol content and for the α-/γ-tocopherol ratio, in a doubled haploid (DH) population of *B. napus* [15]. Recently, about 50 QTL and associated markers for tocopherol content and composition have been detected in another DH population of *B. napus* and its reconstructed F2 [16]. *B. rapa* is also related to the reference plant species, *Arabidopsis thaliana*, in which fourteen QTLs affecting seed tocopherol content and composition have been identified, in two recombinant inbred line (RIL) populations [4]. The genes of the tocopherol synthesis pathways have been identified and cloned by mutation studies and genomic-based approaches in *A. thaliana* and *Synechocystis* sp. PCC 6803. [5,6,14,17–25]. Furthermore, the first *Brassica* gene involved in tocopherol biosynthesis has been cloned, the *B. napus VTE4.a1* gene encoding a γ-tocopherol methyltransferase [26].

Next to tocopherol, there are many more secondary plant metabolites in the plant metabolome that are suggested to have a nutritional effect. Of particular interest are glucosinolates, sulfur containing plant metabolites with anti-carcinogenic properties [27,28] that form a group of more than 100 plant secondary metabolites present primarily in the Brassicaceae family. Each plant species contains a blend of different glucosinolates [29,30]. This blend is largely responsible for the typical flavor and odor of Brassicaceae species plant products. There are significant differences within the Brassicaceae crop species for their glucosinolate profiles [31]. Glucosinolates are grouped into three chemical classes: aliphatic, indolic and aromatic, according to whether their amino acid precursor is methionine, tryptophan or an aromatic amino acid (tyrosine or phenylalanine), respectively [32,33]. Aliphatic glucosinolates are the most prominent glucosinolates found in *Brassica* vegetables [34]. The concentration and chemical structure can vary considerably, depending on the genotype, stage of development, tissue type and environmental conditions [35,36]. More than 90 different aliphatic glucosinolates have been identified among plants [30] of which up to 16 are found in *B. rapa* [37–40]. QTL mapping of leaf aliphatic glucosinolate loci has been carried out in two doubled haploid (DH) populations of *B. rapa*, which identified 16 loci controlling aliphatic glucosinolate concentration [39]. So far, 102 genes putatively involved in glucosinolate biosynthesis have been identified by comparative genomic analyses in *B. rapa* as the orthologues of 52 of such genes in *A. thaliana* [41].

To get unambiguous structural information about a metabolite, Nuclear Magnetic Resonance (NMR), and, particularly, proton NMR (^1^H NMR analysis), probably among the most common methods, as it is a non-destructive method and can simultaneously detect all proton-bearing compounds [42]. Although it has a lower sensitivity compared to Mass Spectrometry (MS) [43], ^1^H NMR spectroscopy has previously been used to uncover qualitative and quantitative differences of various cultivars of *B. rapa*. Different cultivars could be distinguished by elucidated metabolites, for instance, several organic and amino acids, carbohydrates, adenine, indole acetic acid (IAA), phenylpropanoids, flavonoids and glucosinolates [44].

We have used this technique to analyze the genetic variation for a range of (secondary) metabolites in *B. rapa* seedlings of a recently developed RIL population [45]. In addition, a targeted approach, to detect tocopherols, was used to analyze variation for these compounds in seeds of the same population. As the complete genome sequence of *B. rapa* is available [46], our analysis will simplify the identification of candidate genes that can be used for genetic modification or marker-assisted breeding for improved nutritional quality of *B. rapa*.

## 2. Results and Discussion

### 2.1. Seed Tocopherol Concentrations

**Figure 1:**
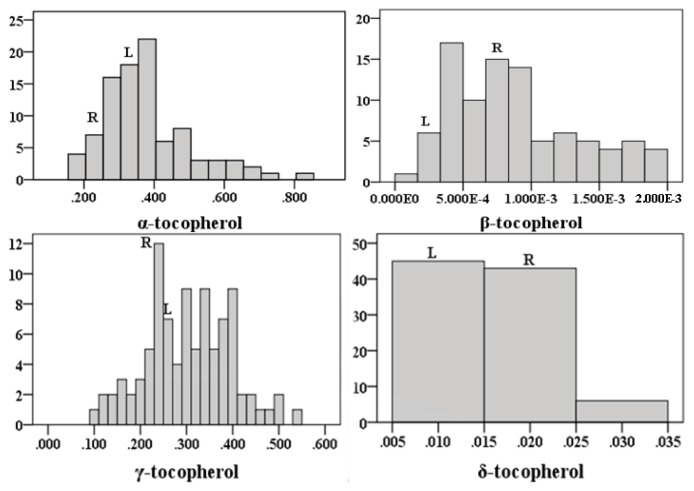

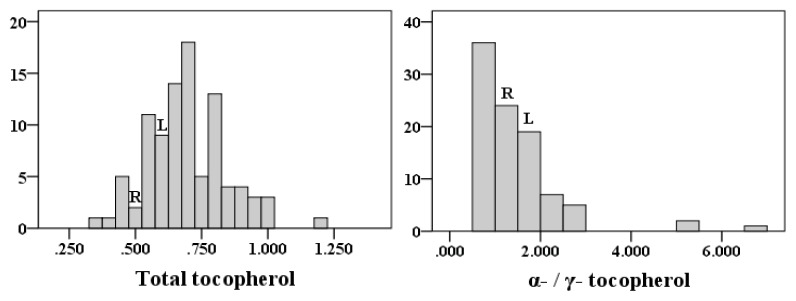
Frequency distributions of non-normalized data of tocopherols in seeds of the L58 × R-o-18 RIL population, including 160 genotypes. The vertical axis indicates the number of lines per trait value class and the horizontal axis the different trait value classes. From left to right and top to the bottom: α-tocopherol (mg/g); β-tocopherol (mg/g); γ-tocopherol (mg/g); δ-tocopherol (mg/g); total tocopherol (mg/g); α/γ tocopherol ratio. The parental values are the mean of three replicates, indicated with L for L58 and R for R-o-18.

We analyzed seeds of the parental lines, L58 and R-o-18, and all individual lines of the L58 × R-o-18 RIL population [45] for tocopherol content (Table 1, Figure 1). L58 showed higher levels than R-o-18 for α-, γ- and total tocopherol. Some lines showed a very high α-tocopherol concentration in comparison to the other components. Transgression beyond the parental values was observed for all measured tocopherols, except δ-tocopherol (Figure 1), suggesting both parents to contain both positive and negative alleles of genes involved in tocopherol biosynthesis. An example of this is the contrasting alleles found at the two major QTLs for α and total tocopherol, respectively, on A6 and A9. This observation also indicates a potential for improvement of vitamin E content and tocopherol composition through classical breeding, by combining both positive alleles in one genotype.

Correlation analysis revealed that total tocopherol concentration was highly positively correlated with the concentrations of α- and γ-tocopherol (Table 2).

### 2.2. QTL Analysis of Seed Tocopherol Concentrations

**Figure 2:**
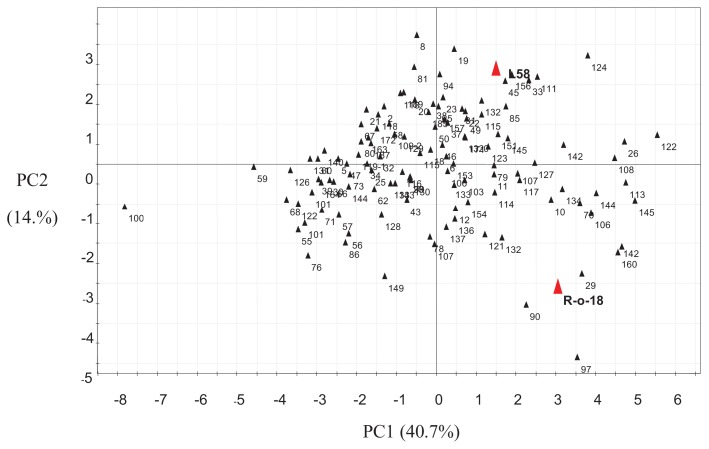
Principal component analysis (PCA) analysis of seedling metabolites based on Nuclear Magnetic Resonance (NMR) signals detected in the L58 × R-o-18 RIL population, including 160 genotypes. Numbers in the figure are line numbers of the RIL population. Parental values are indicated with a red triangle.

Significant variation was observed for all tocopherol components, as indicated by the broad sense heritability (Table 3). Each tocopherol component was subjected to QTL analysis, and QTL related to α-, β-, γ-, δ-, α-/γ- or total tocopherol concentrations were detected on chromosomes A3, A6, A9 and A10 (Table 3, Figure 2). About 45% of the phenotypic variance for α-tocopherol was explained by two QTLs (Al1 and Al2, respectively, on chromosomes A9 and A6). Two QTLs were found for total tocopherol (Toc1 and Toc2), explaining almost 42% of the tocopherol variance. Toc2 co-located with Al1, but Toc1 did not co-locate with Al2, although both mapped to A6. Instead, it co-located with the Ga1 locus for γ-tocopherol. The QTL for δ-tocopherol (De2) mapped to the same region of A9 to which also Al1 and Toc2 were mapped. This region also contains a strong seed coat color QTL (SC1) [45]. The seed color locus, SC1, probably encodes for the *CCR1* gene, a gene involved in lignin biosynthesis [47]. Since there is no reason to suggest a common biochemical basis of biosynthesis of tocopherol and the flavonoids contributing to seed color, a close linkage of different genes, rather than one common gene with pleiotropic effects, is the most likely explanation for this co-location.

As the α-tocopherol concentration is highly positively correlated to the total tocopherol concentration and two of their respective QTLs (Al1 and Toc2) map to the same position, the concentration of α-tocopherol, and not of the intermediate γ-tocopherol, appears to give the major contribution to the overall tocopherol concentration. However, the second Toc locus (Toc1) co-locates with the Ga1 QTL for γ-tocopherol on A6. This means that QTL for both tocopherols with the highest concentrations make a major contribution to the genetic variation for total tocopherol concentrations. The absence of a significant correlation between α-, γ- and δ tocopherol concentrations and the finding that these are controlled by different QTL indicates their independent genetic regulation, which is in agreement with findings of Marwede *et al.* [15] in canola (*B. napus*). Thus, with three independent loci controlling α- and γ-tocopherol, it should be possible to enhance the concentration of both. This will have a negative effect on δ-tocopherol concentration though, since the co-locating De2 and Al1 loci have opposite allele effects. As there are RILs with separated contrasting alleles in this population, we could verify this expectation. The tocopherol analyses of these lines confirm our prediction. A similar antagonistic effect was seen for soybean, where overexpression of the *AtVTE3* gene, encoding the tocopherol biosynthetic enzyme, 2-methyl-6-phytylbenzoquinol methyltransferase, causes a decrease in seed β- and δ-tocopherol with a proportionate increase in α- and γ-tocopherol [5]. The combination of Al1 and Toc2 alleles from the R-o-18 parent leads to the highest tocopherol concentration in this population.

### 2.3. NMR Results of Seedling Metabolites Detection

To further assess the variation in metabolites present in the *B. rapa* RIL population, we performed NMR analysis on young seedlings. Usually, an NMR spectrum consists of hundreds of signals. Among these, 17 compounds in the organic/amino acid, sugar/glucosinolate and aromatic regions of the NMR spectra could be annotated by ^1^H-NMR and confirmed their structures using 2D NMR spectroscopy. ^1^H-NMR data of RIL seedling metabolites were subjected to principal component analysis (PCA) (Figure 2). The score plot of the ^1^H-NMR spectra showed that the two parental lines were quite distinct, especially in principal component 2 (PC2), which was mainly composed of progoitrin, phenylpropanoids and organic compounds. PC1 mostly corresponded to neoglucobrassicin.

L58 had a higher concentration of glucosinolates and phenylpropanoids, whereas the concentrations of sucrose, glucose and glutamate were higher in R-o-18. The major phenylpropanoid was sinapoyl glucose. Correlation analysis showed that the concentrations of several seedling metabolites were highly positively correlated (Table 4).

### 2.4. QTL Analysis of Seedling Metabolites

**Figure 3:**
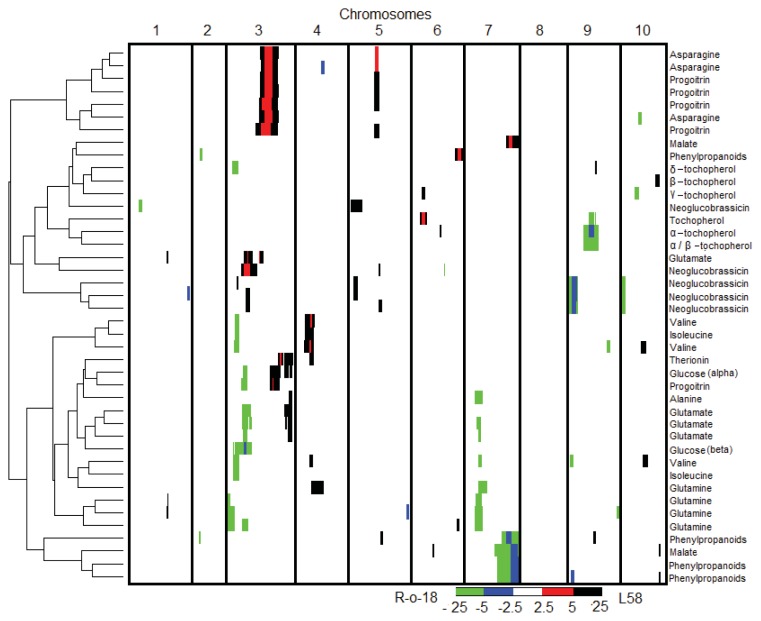
A clustered heat map showing the LOD profiles of QTL identified for the indicated metabolite signals. Columns indicate the 10 *B. rapa* chromosomes, in centimorgans, ascending from the left to right; rows indicate individual compound signal LOD profiles. A color scale is used to indicate the QTL significance corresponding to the LOD score. Positive values (red and black) represent a positive effect on the compound concentration by the L58 allele; negative values (blue and green) represent a positive effect by the R-o-18 allele. The width of a bar indicates the significance interval of the QTL that was calculated by restricted multiple-QTL-model (rMQM) in MAPQTL6. Hierarchical clustering, shown on the left, reflects the correlation between compound concentrations, based on the QTL profiles.

Genetic analysis of 238 signals detected in the NMR spectra enabled the identification of QTL for 146 signals (Table 5, Figure 3). A strong QTL for a compound belonging to the phenylpropanoids was mapped on A7, explaining 43% of the phenotypic variance. Six QTL contributing to variation for alanine, asparagine, glutamine, isoleucine, threonine and valine were detected, explaining up to 37% of the variance. QTL analysis of the glucosinolate NMR signals detected several significant loci, with the most significant one on A9 for neoglucobrassicin. In total, six QTLs for glucosinolates (progoitrin and neoglucobrassicin) were mapped to A3, A5, A9 and A10, with the ones mapping to A3 and A5 possibly co-locating. Previously, five *B. rapa* QTLs related to progoitrin were mapped to chromosomes A1, A3, A4, A8 and A10 [39] using a DH population made from different parents compared to the parents we used to generate the tested RIL population. The authors used forty-day-old leaves for metabolite analysis, while we used young seedlings, which may even still carry glucosinolates originally present in the seed. Therefore, the differences in population and sampled material are considerable, which are the most likely reasons for the differences in detected loci. In any cases, the QTL on A5 and A9 for progoitrin concentration are new loci that have not been reported previously. Extensive studies on aliphatic glucosinolates in *A. thaliana* previously identified genes encoding AOP (2-oxoglutarate-dependent dioxygenase) and MAM (methyl-thioalkylmalate synthase), controlling the modification of side-chain moiety and elongation, respectively, as important factors contributing to genetic variation for glucosinolate concentration and composition [48–52]. The regulation of aliphatic glucosinolate biosynthesis enzymes is controlled in *Arabidopsis* by the R2R3 myb-like transcription factors, MYB28 and MYB29 [53]. The *B. rapa* orthologues of *MYB28* were mapped on A3, A9 and A2, and the orthologues of *MYB29* were mapped on A10 and A3 [41]. The progoitrin QTL presented on A3 with a peak position at 95 cM, co-located with the map positions of *MYB28/MYB29*; there is also a MAM gene in this region.

QTL for the essential amino acids, isoleucine and valine, are co-located on A3 and A4. The isoleucine biosynthesis pathway runs almost parallel to valine biosynthesis, except for its first steps, which involve a threonine deaminase and dehydratase. These loci possibly correspond to the genes encoding the biosynthetic threonine dehydrates (TD) isozyme, similar to what has been isolated from tomato and potato [54,55]. *Arabidopsis* gene AT3G10050, the threonine dehydratase biosynthetic gene, has the syntenic paralog in *B. rapa* on A3, where isoleucine and valine QTL co-located [56]. Non-essential amino acids, such as alanine, asparagine and glutamine, are equally important as the essential amino acids in our body. Eight QTL for non-essential amino acids were identified in this RIL population. These were all independent, except for one QTL on A7, which was shared between alanine and glutamine. At this same region on A7, also, one of the glutamate QTL was mapped. As glutamate is the substrate for glutamine synthesis and the α-amino group of glutamate can be transferred to pyruvate to form alanine [57], this locus is likely to contain a gene involved in the regulation of all three compounds, which is most likely in the upstream, common part of their biosynthesis pathway.

## 3. Experimental Section

### 3.1. Plant Material

The RIL population was derived from a cross between two genotypes belonging to two distinct morphotypes, Cai Xin and Yellow Sarson; both early flowering and self-compatible. The Cai Xin parent is L58, a vegetable type originating from China (*B. rapa* ssp. *parachinensis*). The other parent, R-o-18, is a doubled haploid Yellow Sarson oil type line (*B. rapa* ssp. *trilocularis*) originating from India. This population has been described [45].

### 3.2. Seed Preparation for HPLC

F7 seeds derived from one plant per RIL of the L58 × R-o-18 population were used for tocopherol measurement (two replicate plants per line with two technical replicates from each plant). For the tocopherol extraction, 10–40 mg seeds were ground in 2-mL reaction tubes with a Geno/Grinder 2000 (SPEX-Sample Prep, Metuchen, NJ, USA) using *n*-heptane and 3.0–4.0 mm metal beads. The samples were incubated at −20 °C for 2 h. Further applications and HPLC analyses were performed as described [58–60]. Quantification of the tocopherols was done by fluorescence detection (excitation at λ = 290 nm, emission at λ = 328 nm). To identify the individual tocopherols, the retention times were compared with standard substances from Merck’s tocopherol kit (Merck, Darmstadt, Germany). Total tocopherol content was calculated as the sum of α-, β-, γ- and δ-tocopherol.

### 3.3. Seedling Preparation for NMR Analysis

Thirty seeds per RIL of the *B. rapa* L58 × R-o-18 were used. Seeds were surface sterilized with 70% ethanol (*v*/*v*) for 30 s, followed by agitation for 5 min in sodium hypochlorite (2.0% active chlorite). After three rinses in sterile distilled water, 30 seeds of each individual (for every experiment) were placed in 15 × 90 mm petri dishes, each containing 20–25 mL half strength MS salts and vitamins, without sucrose and solidified with 0.8% (*w*/*v*) agar. Petri dishes were placed vertically in a growth chamber maintained at 25 °C with a 16 h light/8 h dark photoperiod at a light intensity of 60 mEm^−2^ s^−1^. Five-day-old seedlings without roots were harvested and freeze-dried.

20 mg seedlings (dry weight) were extracted with a mixture of 500 μL methanol-*d*_4_ and 500 μL D_2_O (KH_2_PO_4_ buffer, pH 6.0) containing 0.05% TSP (trimethyl silyl propionic acid sodium salt, *w*/*v*) by ultra-sonication for 20 min. After centrifugation, 800 μL supernatant was transferred to an NMR tube. ^1^H NMR spectra were recorded at 25 °C on a 600 MHz Bruker AV600 spectrometer equipped with a cryoprobe, operating at a proton NMR frequency of 600.13 MHz. CD_3_OD was used as the internal lock. Each ^1^H NMR spectrum consisted of 128 scans using the following parameters: TD = 51,200, spectrum width = 16.02 ppm, 0.25 Hz/point, pulse width (PW) = 30° (6.6 μs), acquisition time = 1.70 s. and relaxation delay (RD) = 2.0 s. A pre-saturation sequence was used to suppress the residual H_2_O signal with low power selective irradiation at the H_2_O frequency at μ 4.869 (2915.9 Hz) by 60.59 dB during the recycle delay. Free Induction Decays (FIDs) were Fourier transformed with LB = 0.3 Hz, and the spectra were zero filled to 32 K points. The resulting spectra were manually phased, baseline corrected and calibrated to TMSP at 0.0 ppm, using Topspin (version 2.1, Bruker).

The ^1^H NMR spectra were automatically reduced to an ASCII file. Spectral intensities were scaled to the internal standard (TSP) area and reduced to integrated regions of equal width (0.04 ppm) corresponding to the region of δ 0.3–δ 10.0. The regions of δ 4.75–δ 4.90 and δ 3.28–δ 3.34 were excluded from the analysis, because of the residual signals of HDO and CD_3_OD, respectively. Bucketing was performed by AMIX software (Bruker). Principal component analysis (PCA) was performed with the SIMCA-P software (v. 12.0, Umetrics, Umea, Sweden) with scaling based on the Pareto method.

### 3.4. QTL Analysis

The genetic map was constructed using JoinMap 4.0 [45,61]. MAPQTL6.0 [61] was used for QTL analysis. First, the interval mapping procedure was performed to detect major QTL. For each trait, a 1000× permutation test was performed to calculate the LOD threshold corresponding to a genome-wide false discovery rate of 5% (*p* < 0.05). Markers with LOD scores equal to or exceeding the threshold were used as cofactors in multiple-QTL-model (MQM) mapping. If new QTLs were detected, the linked markers were added to the co-factor list, and the MQM analysis was repeated. If the LOD value of a marker dropped below the threshold in the new model, it was removed from the cofactor list, and the MQM analysis was rerun. This procedure was repeated, until the cofactor list became stable. The final LOD score for each trait was determined by restricted MQM (rMQM) mapping. In some cases, rMQM mapping showed that some cofactors should be on the same linkage group, but at slightly different positions. In that case, the new marker was selected as a cofactor and the whole procedure was repeated.

## 4. Conclusions

The detected genotypic variation in tocopherol seed concentration and seedling metabolites in the RIL population under study allowed the detection of several QTLs for these compounds. The loci we detected can be used to establish diagnostic markers for marker-assisted selection for improved nutritional quality (mainly tocopherol and glucosinolate concentrations). The further analysis of these QTLs affecting metabolic processes will increase our knowledge about the regulatory control of biosynthetic pathways.

## Figures and Tables

**Table 1:** Analysis of tocopherol concentrations in both parents and the L58 × R-o-18 Recombinant Inbred Line (RIL) population, including 160 genotypes. Tocopherol concentration is given in mg per g of seed.

Tocopherol	α-	β-	γ-	δ-	total	α-/γ-ratio
L58 (Mean value ± SE)	0.317 ± 0.006	0.0003 ± 0.0001	0.273 ± 0.003	0.004 ± 0.0004	0.595 ± 0.010	1.159 ± 0.008
R-o-18 (Mean value ± SE)	0.250 ± 0.001	0.0006 ± 0.00008	0.233 ± 0.001	0.019 ± 0.0003	0.502 ± 0.002	1.076 ± 0.003
Max value	0.885	0.0019	0.530	0.034	1.199	8.266
Min value	0.161	0.0001	0.086	0.003	0.325	0.537
Mean value	0.365	0.0008	0.294	0.015	0.675	1.428

**Table 2:** Pearson correlation analysis of tocopherols in the L58 × R-o-18 RIL population, including 160 genotypes.

Trait	α-	β-	γ-	δ-	α-*/*γ-
β-	0.134				
γ-	−0.09	−0.22 *			
δ-	−0.16	0.106	0.15		
α-*/*γ-	0.69 **	0.253 *	−0.65 **	−0.23 *	
Total	0.78 **	−0.024	0.54 **	−0.01	0.19

α-: α-tocopherol; γ-: γ-tocopherol; δ-: δ-tocopherol; Total: total tocopherol; α-/γ-: α-/γ-tocopherol ratio.

** means significant at *p* ≤ 0.01;

* significant at *p* ≤ 0.05.

**Table 3:** Quantitative trait loci (QTL) related to tocopherol concentration in seeds of the *B. rapa* L58 × R-o-18 RIL population, including 160 genotypes. “Peak position” indicates the location of the highest LOD score for each QTL. Flanking markers shows marker names flanking the QTL confidence interval based on a one LOD interval. “% Expl. var.” is the percentage of total phenotypic variance explained by individual QTLs. The allelic effect of each QTL is indicated (effect), which is calculated as μA-μB (μ = mean), where A and B are RILs carrying L58 and R-o-18, respectively, alleles at the relevant QTL position. Effects are given in mg/g or without unit (for the ratio of α/γ tocopherol). H^2^ is broad sense heritability. For all traits, four replicate samples were measured. For β- and δ-tocopherol, values were very small to calculate the difference.

Trait	QTL	Linkage group	LOD	Peak position (cM)	Flanking markers	Confidence interval (cM)	% Expl. var.	Effect	H^2^
α-tocopherol	Al1	A9	10.2	54.4	899051|9912525, E3850M1	51–56.5	34.5	−0.15	0.89
	Al2	A6	3.8	66.9	902204|9940666, 905950|9891649	61–69.6	10.7	+0.08	
β-tocopherol	Be	A10	2.6	80.0	E3851M15, BrID11581	66.8–88	12.1	+0.0004	-
γ-tocopherol	Ga1	A6	4.0	28.4	905326|9911105, 899208|9961195	24–36.5	14.3	+0.08	0.86
	Ga2	A10	3.5	37.3	E3416M30, E3851M1	26–48	12.3	−0.06	
δ-tocopherol	De1	A3	3.0	16.0	E3835M1, VtE	13.5–19	12	−0.004	-
	De2	A9	3.0	63.4	904266|9904851, 902257|9955644	63–65.6	11.5	+0.004	
Total tocopherol	Toc1	A6	8.2	28.4	905326|9911105, 899208|9961195	24–36	27.4	+0.16	0.90
	Toc2	A9	4.9	56.6	E3732M3, 899475|9952883	55–58.8	15.1	−0.12	
α/γ tocopherol ratio	ALGa	A9	5.2	54.4	899051|9912525, E3850M1	51–55.5	22.3	−0.93	0.89

**Table 4:** Pearson correlation analysis of metabolites identified by ^1^H-NMR signals in the L58 × R-o-18 *B. rapa* RIL population, including 160 genotypes. Only significant correlations (at *p* ≤ 0.05) with scores ≥ 0.45 are shown.

Compound signals	Flavonoid3	Neoglucobrassicin	Phenylpropanoid	Glucose alpha	Glucose beta	Progoitrin	Choline	Asparagine	Citrulline	Malate	Valine	Threonine	Alanine	Isoleucine	Glutamine
Neoglucobrassicin	0.53														
Phenylpropanoid	-	-													
Sinapoyl glucose	-	-	0.61												
Glucose alpha	-	-	-												
Glucose beta	-	-	-	0.62											
Progoitrin	-	-	-	0.58	-										
Choline	-	-	-	0.50	0.55	0.68									
Asparagine	-	-	-	-	-	0.95	-								
Citrulline	-	-	-	-	-	-	-	0.56							
Malate	-	0.49	0.66	-	-	0.55	-	-	0.45						
Valine	-	-	-	-	65.0	0.54	0.54	-	-	-					
Threonine	-	-	-	0.56	-	0.46	-	-	-	-	0.48				
Alanine	-	-	-	-	-	0.45	0.54	-	0.52	-	0.80	0.47			
Isoleucine	-	-	-	-	-	-	-	-	0.60	-	0.98	-	0.73		
Glutamine	-	-	-	0.54	0.62	0.58	0.55	-	0.45	-	0.82	0.48	0.75	0.73	
Glutamate	-	-	-	0.68	0.59	0.57	0.57	-	-	0.57	0.84	0.67	0.74	0.69	0.83

**Table 5:** Overview of QTL related to seedling metabolites based on NMR signals detected in the *B. rapa* L58 × R-o-18 RIL population, including 160 genotypes. The peak position indicates the genetic map position of the highest LOD score for each QTL. Flanking markers show marker names flanking the QTL confidence interval based on a one LOD interval. % Expl. var. is the percentage of total phenotypic variance explained by individual QTLs.

Compound signal	Linkage group	LOD	Peak position (cM)	Flanking markers	Confidence interval (cM)	% Expl. Var.
Progoitrin	A3	4.6	24	E3556M6, P2348M160	20.8–30.6	17
	A3	18	95	E3851M18, E3850M2	90–102.5	39

Phenylpropanoids	A5	6	58	P2348M220, 903986|9914577	53–61.5	12
	A2	3	20	BrID101239-A5, E3850M9	16.5–29	9
	A5	4.6	69	E3732M6, 898692|9945263	64.8–71	10
	A6	4.8	101	899475|9952883, P2348M200	92–112	15
	A7	16.5	112	E3416M2, 905396|9906565	110–125	43
	A9	2.8	8	899475|9952883, P2348M200	3–13.4	6

Neoglucobrassicin	A9	6.8	58	BRMS-018-A7, 904702|9902415	55.5–60	15
	A10	2.8	81	E3416M2, 905396|9906565	67–86	6
	A1	3	25	E3851M15, BrID11581	22–36.5	9
	A1	2.8	138	E4051M5, BrID11087	133–140.5	4
	A3	3.3	16	E3835M7, 905044|9901291	12–16.6	6
	A3	8.6	44	E3835M1, VtE	39–47	24
	A5	4.3	18	904714|9903794, BrID101239-A5	14.6–31	14

Alanine	A5	3.8	71	P2348M112, 905015|9915881	68–79	10
	A6	3.4	72	E3851M17, E3416M13	70.6–75	8.5
	A9	24	18	905396|9906565, E3850M4	13–22	50
	A3	3.6	139	P2348M294, E4051M3	137–147.5	12
	A7	4.1	32	BrID10119-A7, P2348M189	30–34.8	13.5

Asparagine	A3	14.5	92	E3851M6, E3851M18	87–94.7	37.3
	A4	3.2	47.7	E3851M9, E3856M3	34–55	6.5
	A5	4.3	58	P2348M220, 903986|9914577	56–60.8	10
	A10	3	48	904135|9928717, P2348M317	37.4–49	7

Glutamine	A1	4.4	83	P2348M66, E3416M8	78–86	8.7
	A3	9.4	19	VtE, P2147M285	16.6–21	23
	A3	7.2	43	E3835M6, 899062|9911548	41.3–47	19
	A4	4	55	E3850M10, E3856M6	47–58.3	12.7
	A5	3	134.5	BrID90357, 902420|9939979	130–138	5.5
	A6	4.1	101	E3732M6, 898692|9945263	92–113	8.3
	A7	7.6	35	901866|9957577, BrID10107-A7	32–47	16
	A9	4.3	107	904535|9904729, 903426|9919397	84–112	8.5

Isoleucine	A3	5.3	22	P2147M276, E3416M14	19–25	15.5
	A4	5.6	34.2	E3851M10, E3850M10	30–48	16.5

Threonine	A3	6	121	902392|9921642, E3835M10	111–125	19.3
	A4	3.2	34.2	E3851M10, E3850M10	31–47.6	10

Valine	A3	6.4	22.5	VtE, P2147M285	19–25	15.3
	A4	8.4	25	E3851M10, E3850M10	13–31	20.3
	A7	3.8	35	901866|9957577, BrID10107-A7	31.5–46.4	8.6
	A9	3.3	0	E3732M12, E3416M2	0–5.5	7.4
	A9	4	84	BrID10187, 904535|9904729	81.5–111	8.5
	A10	4	56	P2348M317, 900140|9938643	52–59	9

Glutamate	A1	5	83	P2348M66, E3416M8	78–91.5	11.2
	A3	9	43	E3416M14, E3835M6	40.6–47	27
	A3	10	110	903854|9907725, P2348M294	106–120	30
	A7	3.6	33.5	BrID10119-A7, P2348M189	30–44.6	10

Malate	A6	4.1	48.5	899015|9918455, 902942|9916766	43–52	10
	A7	11.5	109	BRMS-018-A7, 904702|9902415	105–112.6	33
	A10	3.7	83	BrID11581, E3835M4	78–88.5	9

Glucose (alpha, beta)	A3	5.6	43	E3835M6, 899062|9911548	38–46.4	20
	A3	3.4	95	E3851M18, E3850M2	89–103	13
